# Mutational and phenotypic spectrum of phenylalanine hydroxylase deficiency in Zhejiang Province, China

**DOI:** 10.1038/s41598-018-35373-9

**Published:** 2018-11-20

**Authors:** Ting Chen, Weize Xu, Dingwen Wu, Jiamin Han, Ling Zhu, Fan Tong, Rulai Yang, Zhengyan Zhao, Pingping Jiang, Qiang Shu

**Affiliations:** 1 Division of Medical Genetics and Genomics, The Children’s Hospital, Zhejiang University School of Medicine, Hangzhou, 310058 China; 2 Institute of Genetics and Department of Human Genetics, Zhejiang University School of Medicine, Hangzhou, 310058 China

## Abstract

Phenylalanine hydroxylase deficiency (PAHD), one of the genetic disorders resulting in hyperphenylalaninemia, has a complex phenotype with many variants and genotypes among different populations. Here, we describe the mutational and phenotypic spectrum of PAHD in a cohort of 420 patients from neonatal screening between 1999 and 2016. The observed phenotypes comprised 43.57% classic phenylketonuria, 33.10% mild PKU, and 23.33% mild hyperphenylalaninemia, with an overall PAHD incidence of 1 in 20,445. Genetic testing was performed for 209 patients and 72 variants including seven novel variants were identified. These included two synonymous and five pathogenic nonsynonymous variants (p.S36*, p.T186I, p.L255W, p.F302V and p.R413H). The most common variant among all patients was p.R243Q, followed by p.R241C, p.Y204C, p.R111* and c.442-1G > A. Variants p.R53H and p.F392I occurred only in MHP with 19.3% and 8.0% of the observed alleles respectively. The genotypes p.[R241C];[R243Q], p.[R243Q];[R243Q], and p.[Y204C];[R243Q] were abundant across all PAHD patients. The distributions of the null allele and the three defined genotypes, null/null, null/missense, and missense/missense, were significantly different between the cPKU and mPKU patients. However, no significant differences were found between mPKU and MHP patients, indicating that other modifier factors influence the phenotypic outcome in these patients. The data presented here will provide a valuable tool for improved genetic counseling and management of future cases of PAHD in China.

## Introduction

Phenylalanine hydroxylase deficiency (PAHD), one of the genetic disorders that can lead to hyperphenylalaninemia (HPA), is a phenotypically heterogeneous disorder of phenylalanine (Phe) metabolism. Recessive mutations in *PAH* result in high Phe levels in the blood and brain. More than 1,000 *PAH* mutations have been identified to date, recorded as *PAH*vdb in the Locus-Specific Database (http://www.biopku.org/pah). Undoubtedly, many more variants remain to be detected in different geographic populations. The metabolic phenotypes of PAHD range from mild hyperphenylalaninemia (MHP), which does not require treatment, to classic phenylketonuria (cPKU, OMIM 261600), which requires significant intervention. This broad phenotypic range is due to the different *PAH* genotypes conferring different degrees of reduced enzyme activity. PAHD has an incidence of 1/10,000 in the Europeans population^1^, 1/11,614 in the Chinese population^2^, 1/143,000 in the Japanese population, and a much higher incidence of 1/2,600 in the Turkish population^3^.

Accumulated evidence has demonstrated that the phenotypic and mutational spectrum of PAHD varies among different ethnic and geographic populations. Overall, the two most frequent variants listed in *PAH*vdb are p.R408W (19.2%) and c.1066-11G > A (6.8%). Variants p.R261Q (15.7% of alleles) and p.A403V (11.6% of alleles) are most common in PKU patients from southern Italy^4^, while the most prevalent variant in the Spanish population is p.I65T^5^. In the Chinese population, the highly p.R241C (36%) mutation was detected in Taiwan patients as a founder effect on PAHD^6^, while p.R243Q (17.53%) is common in PKU patients from both northern and southern Mainland China^7^. Additionally, mild PKU (mPKU) and MHP are present with higher incidence in the Chinese population compared with cPKU, which is more prevalent in Eastern Europe^8,9^. *PAH* analysis and phenotype assessment are critical for effective clinical diagnosis and treatment of PAHD, but the parameters of these remain incompletely characterized for both PKU and MHP Chinese patients.

In the present study, we summarized the PAHD mutational and phenotypic spectrum by analyzing *PAH* variants and genotypes in both PKU and MHP patients using samples from neonatal screening for HPA collected between 1999 and 2016 in Zhejiang Province. Seven novel variants of *PAH* were recorded. Heterogeneous genotypes with specific allele frequencies were observed for both PKU and MHP patients.

## Materials and Methods

### Subjects and phenotypic classification

Unrelated samples (n = 420) were collected via the neonatal screening program at the newborn screening center of the Children’s Hospital, Zhejiang University School of Medicine, Zhejiang Province, between 1999 and 2016. The metabolic phenotypes of all subjects were classified solely on the maximum pretreatment plasma Phe levels without dietary therapy as either cPKU (cPKU, ≥1200 μmol/L), mPKU (mPKU, 360–1200 μmol/L), and MHP (120–360 μmol/L) according to our national consensus^10^. Patients with BH4 cofactor deficiency were excluded. Informed consent, blood samples and clinical evaluations were obtained from all participants and families, under protocol approved by the Institutional Review Board of the Children’s Hospital of Zhejiang University School of Medicine.

### *PAH* variant analysis

For the samples collected between 2010 and 2016, two hundred and nine patients consented to taking a gene test. DNA was extracted from peripheral blood using DNA Blood Mini Kit (250) (QIAamp) according to the manufacturer’s protocol. Variants detected by sequencing servicer agency (Biosan, Zhejiang) were further confirmed by Sanger sequencing with 13 pairs of primers (Supplementary Table S1) in an ABI 3700 automated DNA sequencer using the Big Dye Terminator Cycle sequencing reaction kit (Applied Biosystems)^11^. Sequences were aligned with the PAH transcript (NM_000277.2) to identify nucleotide variants. The nomenclature was checked with the Mutalyzer program suite (https://mutalyzer.nl/) and followed the HGVS-nomenclature (http://varnomen.hgvs.org/) for description^12^. After Sanger sequencing, novel missense variants were evaluated with several bioinformatics programs including PolyPhen-2 (http://genetics.bwh.harvard.edu/pph2/), SIFT (http://sift.jcvi.org/), Mutation Assessor (http://mutationassessor.org/), Provean (http://provean.jcvi.org/ index.php), Fathmm (http://fathmm.biocompute.org.uk/), and ANNOVAR (http://wannovar.wglab.org/)^13,14^. Variants predicted to be deleterious based on more than four of the seven parameters were considered putative pathogenic mutations. The annotation version used was *Homo sapiens* GRCh37.

### Genotype analysis

Biallelic mutations were found in 183 cases out of the 209 PAHD patients. According to the *PAH*vdb database, the effects of variants were sorted as synonymous, missense, nonsense, frameshift and splicing site. Null *PAH* alleles are defined as those containing nonsense, deletion, frameshift that result in a truncated PAH protein, missense mutations with <3% enzyme activity *in vitro* compared with the native enzyme, and splice sites^8,15^. Therefore, genotypes were categorized into three defined types in this study: null/null, null/missense, and missense/missense.

### Statistical analyses

Statistical analyses were performed with IBM SPSS Statistics 21. The chi-squared test was used to distinguish between the three genotypes and to analyze the null frequency between the cPKU and mPKU group and between the mPKU and MHP group. A *p* value of <0.01 was considered statistically significant.

## Results

### Clinical phenotype of PAHD patients

A total of 420 patients (206 males and 214 females) were diagnosed with PAHD with an incidence of 1 in 20,445 in Zhejiang Province as part of the NBS program between 1999 and 2016. As shown in Table 1, patient phenotypes were classified as cPKU (183 patients, 43.57%), mPKU (139 patients, 33.10%), or MHP (98 patients, 23.33%). The BIOPKU database (http://www.biopku.org) lists the distribution of these three phenotypes as 54.8%, 27.4%, and 17.8%, respectively^16^. The peak values for pretreatment plasma Phe level varied among the different phenotypes and individual patients with a mean level (µmol/L) of 1,755 for cPKU, 743 for mPKU, and 239 for MHP. No significant correlations were found between phenotype and gender (*p* = 0.89). During follow-up, blood Phe values of three MHP cases were increase to 389 (µmol/L) in one 3-year-old case, 421 (µmol/L) in one 4-year-old case and 367 (µmol/L) in one 6-year-old case respectively after a high protein diet, and then return to normal range by dietary management. The outcomes of mPKU and cPKU patients here with normal treated Phe levels had similar intelligence quotient (IQ) to control individuals throughout their childhood so far.

**Table 1:** Characteristics of PAHD patients enrolled in *PAH* genetic testing.

		PAHD	PKU		MHP
			Classic PKU	Mild PKU	
Patients	Cases	**420**	183	139	98
	Ratio (%)		43.57%	33.10%	23.33%
Gender	Male	206	87	70	49
	Female	214	96	69	49
Peak Phe value	(mean, µmol/L)	1067.1	1755.3	743.0	239.0
Patients who agreed for genetic testing		**209**	80	76	53
Gender	Male	103	38	38	27
	Female	106	42	38	26
Detection rate of alleles		**93**.**80%**	96.30%	97.37%	84.91%
Genotype	Homozygote (%)	14.20%	21.62%	9.59%	8.33%
	Compound heterozygote (%)	**85**.**80%**	78.38%	90.41%	91.67%

### *PAH* variant and allele distributions

**Figure 1:**
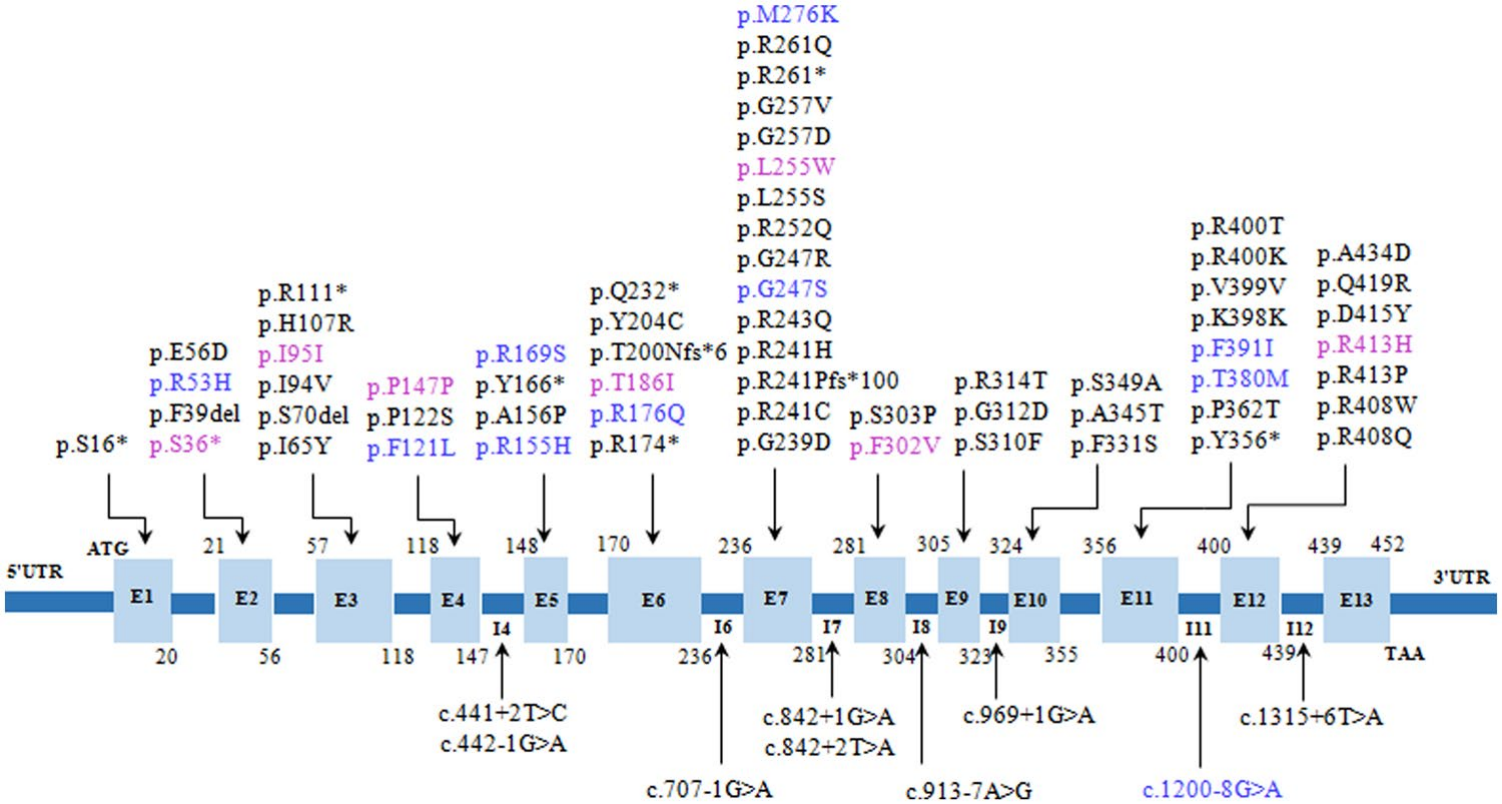
Seventy-two variants were identified across *PAH* for PKU and MHP patients from Zhejiang Province. Purple, novel variants identified in this study; Blue, variants identified in MHP patients only. Amino acid position at the start and end of each exon was labeled numerically.

Genetic testing was performed for 209 patients who consented to. The results indicated 392 different *PAH* alleles with a mean detection rate of 93.80% (Table 1). A total of 72 variants were identified (Table 2), including 46 missense mutations (63.89%), 8 nonsense mutations (11.11%), 10 splice site mutations (13.89%), 4 frameshift mutations (5.56%) resulting from 3 deletions and one duplication, and 4 synonymous variants (5.56%) including the complex one, c.1197A > T (p.V399V)^17^. Ten truncating mutations were produced by 8 nonsense and 2 frameshift mutations (c.598dupA (p.T200Nfs*6) and c.722delG (p.R241Pfs*100)). Variants were unevenly distributed across *PAH* (Fig. 1). There were 63 (87.5%) variants found in the exons, including 16 (22.22%) in exon 7, 8 (11.1%) in exon 11, 7 (9.7%) in exon 12, and 6 (8.3%) each in exons 3 and 6. None were observed in exon 13. It appeared that exons 3, 6, 7, 11, and 12 are hotspot regions for PAHD-associated variants and alleles, consisted with the previous evidence^7^.

**Table 2:** *PAH* variants and allele distribution in PKU and MHP patients from Zhejiang Province.

Index	Nucleotide alteration	Amino acid change	Variant	Region	Alleles in cPKU	Alleles in mPKU	Allele frequency in PKU (%)	Alleles in MHP	Allele frequency in MHP (%)	Allele frequency in PAHD (%)
1	c.47–48delCT	p.S16*	Nonsense	E1	1		0.3			0.26
2	*c*.*107* *C* > *A*	*p*.S36*	Nonsense	E2	1		0.3			0.26
3	c.115_117delTTC	p.F39del	In-frame	E2	1		0.3			0.26
4	**c**.**158** **G** > **A**	**p**.**R53H**	Missense	E2				17	**19**.**3**	**4**.**34**
5	c.168 G > T	p.E56D	Missense	E2	1		0.3			0.26
6	c.194 T > C	p.I65T	Missense	E3	1		0.3			0.26
7	c.208-210delTCT	p.S70del	In-frame	E3	3	1	1.3	2	2.3	1.28
8	c.280 A > G	p.I94V	Missense	E3		1	0.3			0.26
9	*c*.*285* *C* > *T*	*p*.*I95I*	Synonymous	E3	1		0.3			0.26
10	c.320 A > G	p.H107R	Missense	E3		1	0.3			0.26
11	**c**.**331 C > T**	**p**.R111*	Nonsense	E3	13	6	**6**.**3**	2	2.3	**5**.**36**
12	c.361 T > C	p.F121L	Missense	E4				1	1.1	0.26
13	c.364 C > T	p.P122S	Missense	E4	1		0.3			0.26
14	*c*.*441* *T* > *C*	*p*.*P147P*	Synonymous	E4				1	1.1	0.26
15	**c**.**442-1 G > A**		Splice site	I4	12	7	**6**.**3**	3	3.4	**5**.**61**
16	c.441 + 2 T > C		Splice site	I4	2		0.7			0.51
17	c.464 G > A	p.R155H	Missense	E5				1	1.1	0.26
18	c.466 G > C	p.A156P	Missense	E5	1		0.3	1	1.1	0.26
19	c.498 C > G	p.Y166*	Nonsense	E5	2		0.7			0.51
20	c.505 C > A	p.R169S	Missense	E5				2	2.3	0.51
21	c.526 C > T	p.R176*	Nonsense	E6		2	0.7	1	1.1	0.26
22	c.527 G > A	p.R176Q	Missense	E6				1	1.1	0.26
23	*c*.*557* *C* > *T*	*p*.*T186I*	Missense	E6				1	1.1	0.26
24	c.598dupA	p.T200N fs*6	Frameshift	E6		1	0.3			0.26
25	**c**.**611 A > G**	**p**.**Y204C**	Nonsense	E6	18	12	**9**.**9**	5	**5**.**7**	**8**.**93**
26	c.694 C > T	p.Q232*	Nonsense	E6	1		0.3			0.26
27	c.707-1 G > A		Splice site	I6	6	2	2.6	2	2.3	2.55
28	c.716 G > A	p.G239D	Missense	E7	1		0.3			0.26
29	**c**.**721 C > T**	**p**.**R241C**	Missense	E7	5	40	**14**.**8**	7	**8**.**0**	**13**.**27**
30	c.722delG	p.R241Pfs*100	Frameshift	E7	4	2	2.0			1.53
31	c.722 G > A	p.R241H	Missense	E7		1	0.3			0.26
32	**c728G > A**	**p**.**R243Q**	Missense	E7	42	37	**26**.**0**	8	**9**.**1**	**22**.**19**
33	c.739 G > C	p.G247R	Missense	E7		1	0.3			0.26
34	c.739 G > A	p.G247S	Missense	E7				1	1.1	0.26
35	c.740 G > T	p.G247V	Missense	E7		1	0.3			0.26
36	c.755 G > A	p.R252Q	Missense	E7	3	1	1.3	1	1.1	1.28
37	*c*.*764* *T* > *G*	*p*.*L255W*	Missense	E7		1	0.3			0.26
38	c.764 T > C	p.L255S	Missense	E7	1		0.3	1	1.1	0.51
39	c.770 G > T	p.G257V	Missense	E7	3	1	1.3	2	2.3	1.53
40	c.770 G > A	p.G257D	Missense	E7	1		0.3			0.26
41	c.781 C > T	p.R261*	Nonsense	E7		1	0.3	2	2.3	0.77
42	c.782 G > A	p.R261Q	Missense	E7	2	1	1.0			0.77
43	c.827 T > A	p.M276K	Missense	E7				1	1.1	0.26
44	c.842 + 1 G > A		splice site	I7	1		0.3			0.26
45	c.842 + 2 T > A		splice site	I7	1		0.3			0.26
46	*c*.*904* *T* > *G*	*p*.*F302V*	Missense	E8		1	0.3			0.26
47	c.907 T > C	p.S303P	Missense	E8	1		0.3			0.26
48	c.913-7 A > G		splice site	I8		1	0.3	2	2.3	0.77
49	c.929 C > T	p.S310F	Missense	E9		1	0.3			0.26
50	c.935 G > A	p.G312D	Missense	E9		2	0.7			0.51
51	c.940 C > A	p.P314T	Missense	E9	1		0.3	1	1.1	0.51
52	c.969 + 1 G > A		splice site	I9		1	0.3			0.26
53	c.992 T > C	p.F331S	Missense	E10		1	0.3			0.26
54	c.1033 G > A	p.A345T	Missense	E10	1	2	1.0			0.77
55	c.1045 T > G	p.S349A	Missense	E10	1	1	0.7			0.51
56	**c**.**1068 C > A**	**p**.Y356*	Nonsense	E11	11		**3**.**6**	1	1.1	3.06
57	c.1084 C > A	p.P362T	Missense	E11		1	0.3			0.26
58	c.1139 C > T	p.T380M	Missense	E11				1	1.1	0.26
59	**c**.**1174 T > A**	**p**.**F392I**	Missense	**E11**				7	**8**.**0**	1.79
60	c.1194 A > G	p.K398K	Synonymous	E11		1	0.3			0.26
61	**c**.**1197 A > T**	**p**.**V399V**	Complex	**E11**	1	2	1.0			0.77
62	c.1199 G > C	p.R400T	Missense	E11	1		0.3			0.26
63	c.1199 G > A	p.R400K	Missense	E11	1		0.3			0.26
64	c.1200-8 G > A		Splice site	I11				1	1.1	0.26
65	c.1222 C > T	p.R408W	Missense	E12	1		0.3			0.26
66	**c**.**1223** **G** > **A**	**p**.**R408Q**	Missense	E12	4	6	**3**.**3**	1	1.1	2.81
67	c.1238 G > C	p.R413P	Missense	E12	1	2	1.0	2	2.3	1.28
68	*c.1238 G* > *A*	*p.R413H*	Missense	E12	1		0.3	1	1.1	0.51
69	c.1243 G > T	p.D415Y	Missense	E12		1	0.3			0.26
70	c.1256 A > G	p.Q419R	Missense	E12		1	0.3	4	4.5	1.28
71	c.1301 C > A	p.A434D	Missense	E12		1	0.3	2	2.3	0.77
72	c.1315 + 6 T > A		Splice site	I12		5	1.6	2	2.3	1.79
	Amount of Alleles		392		154	150		88		

Italic, novel variants; common mutations are in bold.

Allele frequency in PKU is a ratio as the amount of one variant in cPKU and mPKU to 304 alleles occurred in PKU patients; Allele frequency in MHP is a ratio as the amount of one variant in MHP cases to 88 alleles found in all MHP cases. Allele frequency in PAHD is a ratio as the amount of one variant to all 392 alleles in PAHD.

Among 72 variants, only 12 variants were identified in MHP patients, including p.R53H, p.F121L, p.P147P, p.R155H, p.R169S, p.R176Q, p.T186I, p.G247S, p.M276K, p.T380M, p.F392I, and c.1200-8G > A. Of these, p.R53H and p.F392I were common variants accounting for 19.3% and 8.0% of the alleles, with a predicted residual enzyme activity of 79% (http://www.biopku.org/pah/result-details-pah.asp?ID=668) and 98% (http://www.biopku.org/pah/result-details-pah.asp?ID=811), respectively. Among the 60 variants recorded for the PKU patients, seven variants with relatively high frequencies were p.R243Q, p.R241C, p.Y204C, p.R111*, c.442-1G > A, p.Y356*, and p.R408Q (26.0%, 14.8%, 9.9%, 6.3%, 6.3%, 3.6%, and 3.3%, respectively). These variants were present in approximately 70% of the alleles for PKU. Moreover, eleven variants—p.R243Q, p.R241C, p.Y204C, c.442-1G > A, p.R111*, p.R408Q, c.707-1G > A, p.G257V, p.R413P, p.R252Q, and p.S70del—were observed for all phenotypic groups (i.e., cPKU, mPKU, and MHP).

Seven novel variants were identified (Supplementary Fig. 1.), including two synonymous variants: c.285C > T (p.I95I) in a cPKU patient and c.441T > C (p.P147P) in an MHP patient. Besides the conservation analysis of amino acid, other five pathogenic nonsynonymous variants were evaluated with bioinformatic programs and predicted as putative functional variants (Supplementary Table S2). Of these, c.107C > A (p.S36*) and c.1238G > A (p.R413H) were observed for cPKU; c.764T > G (p.L255W) and c.904T > G (p.F302V) were observed for mPKU; and c.557C > T (p.T186I) was observed for MHP. According to the PAH protein domains, p.S36* variant localized in the regulatory domain, p.T186I, p.L255W and p.F302V in the catalytic domain, and the p.R413H in the oligomerization domain. The pathogenicity of novel variants should be proved by more PAHD cases and their functional analysis *in vitro*.

### Genotypic distribution for PAHD

In total, biallelic mutations were genotyped in 183 individuals, and of which 85.8% (157/183) were compound heterozygous alleles, and 78.38% of these observed for cPKU, 90.28% for mPKU, and 91.89% for MHP patients. Monoallelic mutations were found and confirmed for the remaining 26 patients, of which 16 had the MHP phenotype. There were 112 different genotypic combinations among the PAHD patients including 8 homoallelic genotypes harbored by 26 individuals (Supplementary Table S2): p.[R111*];[R111*], c.[442-1G > A];[442-1G > A], p. [R241Pfs*100];[R241Pfs*100], and p.[Y356*];[Y356*] were carried by cPKU patients only; p.[R243Q];[R243Q] and p.[Y204C];[Y204C] were carried by both cPKU and mPKU patients, p.[R241C];[R241C] was harbored by both mPKU and MHP patients, and p.[R53H];[R53H] was observed for MHP patients only. The most abundant genotypes in the PAHD patients in this study were p.[R241C];[R243Q], p.[R243Q];[R243Q], p.[Y204C];[R243Q], p.[Y204C];[R241C], p.[R111*];[R241C], p.[R111*];[R243Q], [p.R243Q];[c.442-1G > A], p.[R241C];[R241C], p.[Y204C];[Y204C] and p.[R111*];[R261Q], with frequencies of 8.7%, 7.1%, 3.3%, 2.7%, 2.7%, 2.2%, 2.2%, 2.2%, 2.2%, and 1.6%, respectively (Table 3). Strikingly, none of these genotypes were observed to coexist in any of the three phenotypes. However, seven genotypes were concurrent in both cPKU and mPKU patients, and four genotypes associated with not only the mPKU phenotype but also the MHP phenotype (Supplementary Table S3).

**Table 3:** The most prevalent genotypes among the PAHD patients.

	Genotype	AA Change	Phenotype	Frequency^#^
1	c.[331 C > T];[728 G > A]	p.[R111*];[R243Q]	4cPKU	2.2%
2	c.[331 C > T];[782 G > A]	p.[R111^*^];[R261Q]	2cPKU, 1mPKU	1.6%
3	c.[442-1 G > A];[728 G > A]	[c. 442-1 G > A];[p.R243Q]	2cPKU, 2mPKU	2.2%
4	c.[331 C > T];[721 C > T]	p.[R111*];[R241C]	1cPKU, 4mPKU	2.7%
5	c.[611 A > G];[611 A > G]	p. [Y204C];[Y204C]	3cPKU, 1mPKU	2.2%
6	c.[611 A > G];[728 G > A]	p. [Y204C];[R243Q]	4cPKU, 3mPKU	3.3%
7	c.[611 A > G];[721 C > T]	p.[Y204C];[R241C]	1cPKU, 4mPKU	2.7%
8	c.[728 G > A];[728 G > A]	p.[R243Q];[R243Q]	^*^9cPKU, 4mPKU	7.1%
9	c.[721 C > T];[728 G > A]	p.[R241C];[R243Q]	16mPKU	8.7%
10	c.[721 C > T];[721 C > T]	p.[R241C];[R241C]	2mPKU, 2MHP	2.2%

^*^9cPKU,4mPKU: the genotype was distributed in 9 cPKU and 4 mPKU patients.

^#^Frequency, a ratio as the cases of one genotype to the total 183 PAHD patients.

We hypothesized that the genotypes and null alleles would exhibit significant differences in distribution among the three phenotypes. Thus, 29 null *PAH* alleles were sorted into three defined genotype classes (null/null, null/missense, and missense/missense) as described in previous reports (Supplementary Table S4)^8,18^. Of the cPKU patients, 77.03% carried at least one null allele, with 54.79% observed for mPKU patients. Furthermore, the null/null genotype was identified in 26 cPKU patients, but only in six mPKU patients (Table 4). The distribution of the three defined genotypes, null/null, null/missense, and missense/missense, were significantly different (*p* < 0.001) between cPKU and mPKU patients. The null allele frequency also differed remarkably between cPKU and mPKU (*p* < 0.001). No significant difference was found between mPKU and MHP patients in either the distribution of genotypes or the null allelic frequency (*p* = 0.21 and *p* = 0.57, respectively).

**Table 4:** The three classes of defined genotypes and null allele frequencies associated with the PAHD phenotypes.

Phenotype	Defined Genotypes			*p* value	Patients with null allele (%)	Frequency of null allele (%)	*p* value
	null/null	null/missense	missense/missense				
Classic PKU	26	31	17	*p1* < 0.001	77.03	56.08	*p1* < 0.001
Mild PKU	6	34	33		54.79	31.51	
MHP	0	20	16	*p2* = 0.21	55.56	27.78	*p2* = 0.57

*p1*, difference between cPKU and mPKU patients; *p2*, difference between mPKU and MHP.

3 × 2 contingency analysis for genotype, 2 × 2 contingency analysis for allele frequencies.

## Discussion

Here, we carried out a retrospective study on samples obtained via neonatal screening for HPA since 1999 in Zhejiang Province of southeast China, illustrating the mutational and phenotypic spectrum of PAHD for a greater understanding of *PAH* gene variants and their association with PAHD phenotypes.

It is confirmed that the incidence of PAHD for south China is lower than that for Northern China alone (1/3,425–1/7,849)^19,20^. Compared with the data from the BIOPKU database, the comprising of the three PAHD phenotypes in the present study is different that more than 50% of PAHD cohort from Zhejiang Province comprises mPKU and MHP patients as reported in Denmark PAHD group^20^.

The identified variants included 63.89% missense mutations, 13.89% splice site mutations, and 11.11% nonsense mutations as previously observed^21^. Exons 3, 6, 7, 11, and 12 appeared to be hotspots for PAHD-associated variants and alleles, which was consistent with previous reports for Chinese and Asian populations^7,22–24^. However, phenotype frequency depends on the allele frequency of particular *PAH* variants. Among the PKU patients, 70% of the allele frequency was contributed by only 7 mutations. The most frequent allele was p.R243Q (22.19%), followed by p.R241C (13.27%), p.Y204C (8.93%), p.R111*(5.36%), c.442-1G > A (5.61%), p.Y356* (3.06%), and p.R408Q (2.81%), but excluded the p.V399V and p.R413P mutations which common in Northern China^22^. Notably, p.R241C seemed to be present with higher incidence in mPKU and MHP patients from the south of China than that described for other regions, which may be represent the expansion or migration of the certain populations^7,11^. The classic PKU mutation c.1222C > T (p.R408W), the most frequent allele in Eastern Europe, was carried by only one cPKU patient in our cohort who also had a 1,706 µmol/L maximum pretreatment Phe level. The p.R53H and p.F392I variants observed for MHP patients with a Phe value of <360 (µmol/L) were present in 27% of the alleles and 49% of the MHP patients (Table 2). While p.R53H is known to be harbored by Japanese PKU patients^25^, it is more common in the general Korean population with a frequency of 2.57%^26,27^. Since these two mutations are observed in healthy subjects and they retain >70% residual PAH activity, p.R53H and p.F392I are classified here as mutations associated with MHP. Another notable finding was that 30% of the MHP patients (16/53) with abnormal Phe values observed during newborn screening were carriers of a pathogenic allele. Genetic counseling should be provided to such patients prior to conception.

The genotypes in our PAHD cohort were highly heterogeneous as >60% of the patients presented with a unique genotype, and 85.8% were compound heterozygotes compared with 76% reported in the BIOPKU database^16^. The p.[R241C];[R243Q] (8.7%) genotype was the most prevalent, followed by p.[R243Q];[R243Q] (7.1%), p.[Y204C];[R243Q] (3.3%), p.[Y204C];[R241C] (2.7%), and p.[R111*];[R241C] (2.7%), which is in accordance with earlier findings that p.[R243Q];[R243Q], p.[Y204C];[R243Q], and p.[R241C];[R243Q] constitute the major genotypes in southern China^8^. Interestingly, two p.R241C homozygous patients showed MHP, which was also detected in Korean PAHD population before^23^. The distribution of null alleles and the genotypes null/null, null/missense, and missense/missense showed significant differences between cPKU and mPKU patients as well as between cPKU and MHP patients. However, no significant differences were observed for the distribution of either between mPKU and MHP patients. This indicates that other modifier factors may influence PAHD phenotype, especially for mPKU and MHP. It is therefore unsurprising that several genotypes are shared by both mPKU and MHP phenotypes.

Certainly, the specific PAH genotype is key for determining the metabolic phenotype. More than 80% (26/32) of the double-null genotypes correlated to cPKU phenotype in the present study. Interestingly, MHP and mPKU phenotypes have similar frequencies of null alleles in genotypes. This implies that the interaction between compound heterozygous alleles play a major role in phenotypic outcome^28^. Additionally, we confirmed that in MHP patients, mild mutations determine disease severity. Genotypes comprising a combination of p.R58H with one of the null alleles p.R111*, c.442-1G > A, p.Y204C, p.Y356*, or p.L255S, resulted in the MHP phenotype. A similar phenomenon was observed for the p.F392I/null genotype associated with the MHP phenotype, although further samples are needed to confirm this, and the effect on PAH structure and function needs to be considered for further insights. In conclusion, the data presented in this study will provide a valuable tool for improved genetic counseling and management of future patients of PAHD in China.

## Electronic supplementary material

Supplementary TableS1,S2,S3,S4 & Figure 1

## References

[1] Steinfeld R, Kohlschutter A, Ullrich K, Lukacs Z (2004). Efficiency of long-term tetrahydrobiopterin monotherapy in phenylketonuria. J. Inherit. Metab. Dis..

[2] Shi XT (2012). Newborn screening for inborn errors of metabolism in mainland china: 30 years of experience. JIMD Rep.

[3] Mitchell J, Trakadis YJ, Scriver CR (2011). Phenylalanine hydroxylase deficiency. Genet Med..

[4] Daniele A (2007). Molecular epidemiology of phenylalanine hydroxylase deficiency in Southern Italy: a 96% detection rate with ten novel mutations. Ann Hum Genet..

[5] Desviat LR (2004). Tetrahydrobiopterin responsiveness: results ofBH4 loading test in 31 Spanish PKU patients and correlation with their genotype. Mol Genet Metab.

[6] Chien YH (2004). Mutation spectrum in Taiwanese patients with phenylalanine hydroxylase deficiency and a founder effect for the R241C mutation. Hum Mutat.

[7] Li N (2015). Molecular characterisation of phenylketonuria in a Chinese mainland population using next generation sequencing. Sci Rep..

[8] Zhu T (2013). Variations in genotype-phenotype correlations in phenylalanine hydroxylase deficiency in Chinese Han population. Gene.

[9] Blau N (2016). Genetic of phenylketonuria: then and now. Hum Mutat..

[10] Yang Y, Ye J (2014). Subspecial Group of Endocrine, Hereditary and Metabolic Diseases; Society of Pediatrics, Chinese Medical Association; Newborn Screening Committee of Professional Society of Birth Defect Prevention and Control; & Chinese Association of Preventive Medical. Consensus about the diagnosis and treatment of hyperphenylalaninemia [Article in Chinese]. Zhonghua Er Ke Za Zhi..

[11] Zhu T (2010). Mutational spectrum of phenylketonuria in the Chinese Han population: a novel insight into the geographic distribution of the common mutations. Pediatr Res..

[12] den Dunnen JT (2016). HGVS Recommendations for the Description of Sequence Variants 2016 Update. Hum Mutat..

[13] Wang K, Li M, Hakonarson H (2010). ANNOVAR: functional annotation of genetic variants from high-throughput sequencing data. Nucleic Acids Research.

[14] Chang X, Wang K (2012). wANNOVAR: annotating genetic variants for personal genomes via the web. J Med Genet..

[15] Tao J (2015). Correlation between genotype and the tetrahydrobiopterin- responsive phenotype in Chinese patients with phenylketonuria. Pediatr Res..

[16] Wettstein S (2015). Linking genotypes database with locus-specific database and genotype-phenotype correlation in phenylketonuria. Eur J Hum Genet.

[17] Chao HK, Hsiao KJ, Su TS (2001). A silent mutation induces exon skipping in the phenylalanine hydroxylase gene in phenylketonuria. Hun Genet..

[18] Blau N, Shen N, Carducci C (2014). Molecular genetics and diagnosis of phenylketonuria: state of the art. Expert. Rev. Mol. Diagn..

[19] Gu XF, Wang ZG (2004). Screening for phenylketonuria and congenital hypothyroidism in 5.8 million neonates in China. Zhonghua Yu Fang Yi Xue Za Zhi..

[20] Jiang QF (2013). Screening analysis of neonatal congenital hypothyroidism and phenylketonuria in Guilin city. Int J Lab Med..

[21] Bayat A, Yasmeen S, Lund A, Nielsen JB, Møller LB (2016). Mutational and phenotypical spectrum of phenylalanine hydroxylase deficiency in Denmark. Clin Genet.

[22] Liu N (2017). Spectrum of *PAH* gene variants among a populatin of Han Chinese patients with phenylketonuria from northern China. BMC Med Genet..

[23] Okano Y (1998). Molecular characterization of phenylketonuria in Japanese patients. Hum Genet.

[24] Lee DH (2004). The molecular basis of phenylketonuria in Koreans. J Hum Genet..

[25] Okano Y, Kudo S, Nishi Y, Sakaguchi T, Aso K (2011). Molecular characterization of phenylketonuria and tetrahydrobiopterin-responsive phenylalanine hydroxylase deficiency in Japan. J Hum Genet..

[26] Choi R (2017). Reassessing the significance of the PAH c.158G>A (p.Arg53His) variant in patients with hyperphenylalaninemia. J Pediatr Endocrinol Metab..

[27] Park, Y. S. *et al*. Identification of three novel mutations in Korean phenylketonuria patients: R53H, N207D, and Y325X. *Hum mutat* Suppl, S121–122 (1998). 10.1002/humu.1380110140 9452061

[28] Arturo EC (2016). First structure of full-length mammalian phenylalanine hydroxylase reveals the architecture of an autoinhibited tetramer. Proc Natl Acad Sci USA.

