# Supplementary material for: Mutational and phenotypic spectrum of phenylalanine hydroxylase deficiency in Zhejiang Province, China
Source: Sci Rep. 2018 Nov 20;8:17137. doi: 10.1038/s41598-018-35373-9 (PMC6244417; doi:10.1038/s41598-018-35373-9)
Supplement: Supplementary file 1 — Supplementary TableS1,S2,S3,S4 & Figure 1 [file 41598_2018_35373_MOESM1_ESM.pdf]

MS#: SREP-18-09010C

Mutational and phenotypic spectrum of phenylalanine hydroxylase deficiency in Zhejiang Province, China

Ting Chen[1,2#], Weize Xu[1#], Dingwen Wu[1], Jiamin Han[2],Ling Zhu[1], Fan Tong[1], Rulai Yang[1], Zhengyan Zhao[1], Pingping Jiang[1,2*], Qiang Shu[1*]

Supplementary Table S1 Primers for *PAH* sequencing

Code	Primers (5' to 3')
1F	TTAAAACCTTCAGCCCCACG
1R	TGGAGGCCCAAATTCCCCTAACTG
2F	GAGGTTTAACAGGAATGAATTGCT
2R	TCCTGTGTTCTTTTCATTGC
3F	GCCTGCGTTAGTTCCAGTGA
3R	CTTATGTTGCAAAATTCCTC
4F	GTTCTGCCAATCTGTACTCAGG
4R	GTAGAGAAGGTAAGAGGAAGGG
5F	GGGAAGGAGACATGCACTGTCATGG
5R	AACTGGATGAGGGCAAGGGAGAAGC
6F	CACAGGTTCTGGTCCCCGAC
6R	CTCTCCTCTGCCTCAATCCTC
7F	GGTGATGAGCTTTGAGTTTTCTTTC
7R	AGCAATGAACCCAAACCTC
8F	GCCCTACCCTGCACCTGTCTCT
8R	CCTCCCTGGGCTCAACTCATT
9F	ACACTCAGGGTCTATGTGGG
9R	CAAGGGTTTTCAAGGGCTAC
10F	TCCCCTCCAGAAACACCCTCAT
10R	ACCCACAGCCATCATCAAATCAT
11F	TGGGCTGTGATGTAGAAGGAAT
11R	CGGAAGAAAAGGAGGGTGGAG
12F	ATGCCACTGAGAACTCTCTT
12R	AGTCTTCGATTACTGAGAAA
13F	CCCTGGGAAGTGACCTATGG
13R	TTTGCTTTTCGGACTTTTTCTG

Supplementary Table S2 Predicted functional effect of novel variants

Index	Nucleotide alteration	AA change	Variant	SIFT	Polyphen-2	Mutation Assessor	LRT	FATHMM	PROVEAN	MetaSVM
1	c.107C>A	p.S36*	nonsynonymous	D	D	D	D	D	D	D
2	c.557C>T	p.T186I	nonsynonymous	D	D	D	D	D	D	D
3	c.764T>G	p.L255W	nonsynonymous	D	D	A	D	D	D	D
4	c.904T>G	p.F302V	nonsynonymous	D	D	D	D	D	D	D
5	c.1238G>A	p.R413H	nonsynonymous	D	B	D	D	D	D	D

A, Automatically disease-causing; B, Benign; D, Deleterious or Damaging; N, Neutral; T, Tolerated.

Supplementary Table S3 The 112 genotypes harbored by the biallelic PAHD patients.

Index	Genotype	AA Change	Defined Genotype	Phe (μmol/L)	cPKU	mPKU	MHP	Frequency* (%)
1	c.[107C>A];[728G>A]	p.[S35*];[R243Q]	null/missense	1980	1			0.55
2	c.[115_117delTTC];[611A>G]	p.[F39del];[Y204C]	null/null	1340	1			0.55
3	c.[158G>A];[158G>A]	p.[R53H];[R53H]	missense/missense	132			1	0.55
4	c.[158G>A];[331C>T]	p.[R53H];[R111*]	null/missense	258			1	0.55
5	c.[158G>A];[441A>G]	p.[R53H];[P147P]	missense/?	141			1	0.55
6	c.[158G>A];[442-1G>A]	[p.R53H]; [c.442-1G>A]	null/missense	265			1	0.55
7	c.[158G>A];[611A>G]	p.[R53H];[Y204C]	null/missense	162			1	0.55
8	c.[158G>A];[728G>A]	p.[R53H];[R243Q]	missense/missense	213			2	1.1
9	c.[158G>A];[755G>A]	p.[R53H];[R252Q]	null/missense	292			1	0.55
10	c.[158G>A];[764T>C]	p.[R53H];[L255S]	null/missense	141			1	0.55
11	c.[158G>A];[913-7A>G]	[p.R53H]; [c.913-7A>G]	null/missense	202			1	0.55
12	c.[158G>A];[1068C>A]	p.[R53H];[Y356*]	null/missense	169			1	0.55
13	c.[158G>A];[1238G>C]	p.[R53H];[R413P]	missense/missense	191			1	0.55
14	c.[168G>T];[728G>A]	p.[E56D];[R243Q]	missense/missense	1592	1			0.55
15	c.[194T>C];[1068C>A]	p.[I65T];[Y356*]	null/missense	1861	1			0.55
16	c.[208-210delTCT];[466G>C]	p.[S70del];[A156P]	null/missense	256			1	0.55
17	c.[208-210delTCT];[755G>A]	p.[S70del];[R252Q]	null/null	1160		1		0.55
18	c.[208-210delTCT];[940C>A]	p.[S70del];[P314T]	null/missense	148			1	0.55
19	c.[280A>G];[722delG]	p.[I94V];[R241Pfs*100]	null/missense	632		1		0.55
20	c.[286G>A];[611A>G]	p.[I95I];[Y204C]	null/?	1867	1			0.55
21	c.[320A>G];[331C>T]	p.[H107R];[R111*]	null/missense	392		1		0.55
22	c.[331C>T];[331C>T]	p.[R111*];[R111*]	null/null	1562	1			0.55
23	c.[331C>T];[441+2T>C]	[p.R111*];[c.441+2T>C]	null/null	2890	1			0.55
24	c.[331C>T];[557C>T]	p.[R111*];[T186I]	null/missense	164			1	0.55
25	c.[331C>T];[707-1G>A]	[p.R111*];[c.707-1G>A]	null/null	2319	1			0.55
26	c.[331C>T];[721C>T]	p.[R111*];[R241C]	null/missense	402-1965	1	4		**2.7**
27	c.[331C>T];[728G>A]	p.[R111*];[R243Q]	null/missense	1638-3070	4			**2.2**
28	c.[331C>T]:[770G>T]	p.[R111*];[G257V]	null/null	2319	1			0.55
29	c.[331C>T];[782G>A]	p.[R111*];[R261Q]	null/missense	1163-1974	2	1		1.6
30	c.[331C>T];[1068C>A]	p.[R111*];[Y356*]	null/null	2303	1			0.55
31	c.[441+2T>C];[907T>C]	[c.441-1G>A];[p.F303P]	null/missense	1918	1			0.55
32	c.[442-1G>A];[47-48delCT]	[c.441-1G>A];[p.S16*]	null/null	2453	1			0.55
33	c.[442-1G>A];[442-1G>A]		null/null	2664	1			0.55
34	c.[442-1G>A];[498C>G]	[c.441-1G>A];[p.Y166*]	null/null	1834	1			0.55
35	c.[442-1G>A];[611A>G]	[c.441-1G>A];[p.Y204C]	null/null	1350	1			0.55
36	c.[442-1G>A];[694C>T]	[c.441-1G>A];[p.Q232*]	null/null	1642	1			0.55
37	c.[442-1G>A];[721C>T]	[c.441-1G>A];[p.R241C]	null/missense	709-724		2		1.1
38	c.[442-1G>A];[728G>A]	[c.441-1G>A];[p.R243Q]	null/missense	706-1597	2	2		**2.2**
39	c.[442-1G>A];[770G>A]	[c.441-1G>A];[p.G257D]	null/missense	1531	1			0.55
40	c.[442-1G>A];[1068C>A]	[c.441-1G>A];[p.Y356*]	null/null	2168	1			0.55
41	c.[442-1G>A];[1084C>A]	[c.441-1G>A];[p.P362T]	null/missense	771		1		0.55
42	c.[442-1G>A];[1174T>A]	[c.441-1G>A];[p.F392I]	null/missense	344			1	0.55

43	c.[442-1G>A];[1222C>T]	[c.441-1G>A];[p.R408W]	null/null	1706	1			0.55
44	c.[442-1G>A];[1238G>C]	[c.442-1G>A];[p.R413P]	null/missense	3539	1			0.55
45	c.[442-1G>A];[1315+6T>A]		null/null	421-520		2		1.1
46	c.[464G>A];[913-7A>G]	[p.R155H];[c.913-7A>G]	null/missense	300			1	0.55
47	c.[466G>C];[770G>T]	p.[A156P];[G257V]	null/null	1531	1			0.55
48	c.[498C>G];[611A>G]	p.[Y166*];[Y204C]	null/null	1907	1			0.55
49	c.[505C>A];[1200-8G>A]	p.[R169S];[c.1200-8G>A]	null/missense	196			1	0.55
50	c.[526C>T];[721C>T]	p.[R176*];[R241C]	null/missense	571-808		2		1.1
51	c.[598dupA];[728G>A]	p.[T200N fs*6];[R243Q]	null/missense	696		1		0.55
52	c.[611A>G];[611A>G]	p. [Y204C]; [Y204C]	null/null	681-2370	3	1		**2.2**
53	c.[611A>G];[707-1G>A]	[p.Y204C];[c.707-1G>A].	null/null	1432-1691	2			1.1
54	c.[611A>G];[721C>T]	p. [Y204C];[R241C]	null/missense	451-1450	1	4		**2.7**
55	c.[611A>G];[722delG]	p. [Y204C];[R241Pfs*100]	null/null	1615	1			0.55
56	c.[611A>G];[728G>A]	p. [Y204C];[R243Q]	null/missense	816-2453	4	3		**3.8**
57	c.[611A>G];[1174T>A]	p. [Y204C];[F392I]	null/missense	253			1	0.55
58	c.[611A>G];[1197A>T]	p.[Y204C];[V399V]	null/null	425		1		0.55
59	c.[611A>G];[1223G>A]	p.[Y204C];[R408Q]	null/missense	870-1378	1	1		1.1
60	c.[611A>G];[1243G>T]	p.[Y204C];[D415Y]	null/missense	477		1		0.55
61	c.[611A>G];[1256A>G]	p.[Y204C];[Q419R]	null/missense	176			1	0.55
62	c.[707-1G>A];[721C>T]	p.[IVS6-1G>A];[R241C]	null/missense	895		1		0.55
63	c.[707-1G>A];[1033G>A]	p.[IVS6-1G>A];[A345T]	null/missense	889-1371	1	1		1.1
64	c.[707-1G>A];[1223G>A]	p.[IVS6-1G>A];[R408Q]	null/missense	1860	1			0.55
65	c.[716G>A];[728G>A]	p.[G239D];[R243Q]	missense/missense	1323	1			0.55
66	c.[721C>T];[361T>C]	p.[R241C];[F121L]	missense/missense	317			1	0.55
67	c.[721C>T];[721C>T]	p.[R241C];[R241C]	missense/missense	255-613		2	2	**2.2**
68	c.[721C>T];[728G>A]	p.[R241C];[R243Q]	missense/missense	387-970		16		**8.7**
69	c.[721C>T];[764T>G]	p.[R241C];[L255W]	missense/missense	1043		1		0.55
70	c.[721C>T];[770G>T]	p.[R241C];[G257V]	null/missense	532		1		0.55
71	c.[721C>T];[913-7A>G]	[p.R241C];[c.913-7A>G]	null/missense	764		1		0.55
72	c.[721C>T];[935G>A]	p.[R241C];[G312D]	missense/missense	615-683		2		1.1
73	c.[721C>T];[969+1G>A]	[p.R241C];[c.969+1G>A]	null/missense	553		1		0.55
74	c.[721C>T];[1068C>A]	p.[R241C];[Y356*]	null/missense	1280	1			0.55
75	c.[721C>T];[1139C>T]	p.[R241C];[T380M]	missense/missense	164			1	0.55
76	c.[721C>T];[1223G>A]	p.[R241C];[R408Q]	missense/missense	1245	1			0.55
77	c.[721C>T];[1238G>A]	p.[R241C];[R413H]	missense/missense	865		1		0.55
78	c.[721C>T];[1301C>A]	p.[R241C];[A434D]	null/missense	271-371		1	1	1.1
79	c.[722delG];[722delG]	p.[R241Pfs*100];[R241Pfs*100]	null/null	2235	1			0.55
80	c.[722delG];[1223G>A]	p.[R241Pfs*100];[R408Q]	null/missense	1053-1556	1	1		1.1
81	c.[728G>A];[364C>T]	p.[R243Q];[P122S]	missense/missense	2575	1			0.55
82	c.[728G>A];[527G>A]	p.[R243Q];[R176Q]	missense/missense	248			1	0.55
83	c.[728G>A];[707-1G>A]	[p.R243Q];[c.707-1G>A]	null/missense	1597	1			0.55
84	c.[728G>A];[728G>A]	p.[R243Q];[R243Q]	missense/missense	432-2495	9	4		**7.1**
85	c.[728G>A];[755G>A]	p.[R243Q];[R252Q]	null/missense	1908	1			0.55
86	c.[728G>A];[940C>A]	p.[R243Q];[P314T]	missense/missense	1561	1			0.55

87	c.[728G>A];[1045T>G]	p.[R243Q];[S349A]	missense/missense	977-1840	1	1		1.1
88	c.[728G>A];[1068C>A]	p.[R243Q];[Y356*]	null/missense	1970-2168	2			1.1
89	c.[728G>A];[1174T>A]	p.[R243Q];[F391I]	missense/missense	238			1	0.55
90	c.[728G>A];[1194A>G]	p.[R243Q];[K398K]	missense/missense	840		1		0.55
91	c.[728G>A];[1197A>T]	p.[R243Q];[V399V]	null/missense	1907	1			0.55
92	c.[728G>A];[1199G>C]	p.[R243Q];[R400T]	missense/missense	1440	1			0.55
93	c.[728G>A];[1223G>A]	p.[R243Q];[R408Q]	missense/missense	603-828		3		1.6
94	c.[728G>A];[1238G>C]	p.[R243Q];[R413P]	missense/missense	1492	1			0.55
95	c.[728G>A];[1238G>A]	p.[R243Q];[R413H]	missense/missense	187			1	0.55
96	c.[728G>A];[1256A>G]	p.[R243Q];[Q419R]	missense/missense	200			1	0.55
97	c.[728G>A];[1315+6T>A]	[p.R243Q];[c.1315+6T>A]	null/missense	293-869		1	1	1.1
98	c.[739G>C];[992T>C]	p.[G247R];[F331S]	missense/missense	645		1		0.55
99	c.[739G>A];[1256A>G]	p.[G247R];[Q419R]	missense/missense	188			1	0.55
100	c.[740G>T];[1238G>C]	p.[G247V];[R413P]	null/missense	1137		1		0.55
101	c.[755G>A];[764T>C]	p.[R252Q];[L255S]	null/null	2446	1			0.55
102	c.[755G>A];[1199G>A]	p.[R252Q];[R400K]	null/missense	1716	1			0.55
103	c.[770G>T];[1174T>A]	p.[G257V];[F392I]	null/missense	153-299			2	1.1
104	c.[781C>T];[1174T>A]	p.[R261*];[F392I]	null/missense	272			1	0.55
105	c.[781C>T];[1223G>A]	p.[R261*];[R408Q]	null/missense	672		1		0.55
106	c.[827T>A];[1223G>A]	p.[M276K];[R408Q]	missense/missense	349			1	0.55
107	c.[842+1G>A];[1068C>A]	[c.842+1G>A];[p.Y356*]	null/null	1902	1			0.55
108	c.[842+2T>A];[1068C>A]	[c.842+2T>A];[p.Y356*]	null/null	1630	1			0.55
109	c.[929C>T];[904T>G]	p.[S310F];[F302V]	missense/missense	464		1		0.55
110	c.[1068C>A];[1068C>A]	p.[Y356*];[Y356*]	null/null	1640	1			0.55
111	c.[1197A>T];[1315+6T>A]	[p.V399V];[c.1315+6T>A]	null/null	368		1		0.55
112	c.[1238G>C];[1256A>G]	p.[R413P];[Q419R]	missense/missense	208			1	0.55

*Frequency, percentage for the cases shared same one genotype in 183 PAHD individuals.

Supplementary Table S4 Classification of the 29 null mutations by type

**Mutation type**	**Mutations**	**Number**
Truncation (by substitution, deletion, or frameshift)	p.S16*, p.S36*, p.R111*, p.Y166*, p.R176*, p.Q232*, p.T200N fs*6, p.R241Pfs*100, p.R261*, p.Y356*	10
Splice site	c.442-1G>A, c.441+2T>C, p.Y204C, c.707-1G>A, c.842+1G>A, c.842+2T>A, c.913-7A>G, c.969+1G>A, c.1200-8G>A, c.1315+6T>A, p.V399V	11
Deletion (in-frame)	p.S70del, p.F39del	2
Missense (residual PAH activity <3%)	p.G247V, p.R252Q, p.L255S,p. G257V, p.R408W, p. A434D	6

Supplementary Figure 1. Seven novel variants of *PAH.* (a) Partial sequence chromatograms of seven novel variants. **(b)** Conservation of amino acid for five nonsynonymous variants in species, *Homo sapiens*: NP_000268.1; *Pan troglodytes*: XP_001156919.1; *Bos Taurus*: NP_001039523.1; *Mus musculus*: NP_032803.2; *Rattus norvegicus*: NP_036751.2; *Gallus gallus*: NP_001001298.1; and *Danio rerio*: NP_956845.1. The arrow indicates the location of the changed nucleotide or amino acid.

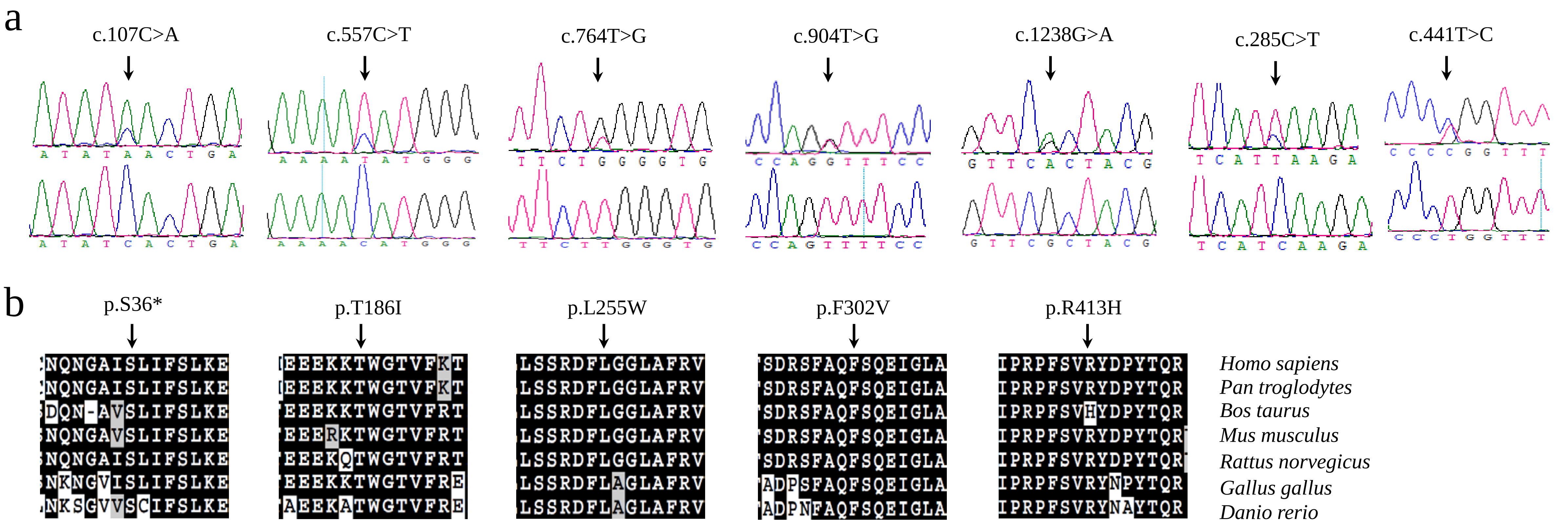

Supplementary Figure 1. Seven novel variants of *PAH*. (a) Partial sequence chromatograms of seven novel variants. **(b)** Conservation of amino acid for five nonsynonymous variants in species, *Homo sapiens*: NP_000268.1; *Pan troglodytes*: XP_001156919.1; *Bos Taurus*: NP_001039523.1; *Mus musculus*: NP_032803.2; *Rattus norvegicus*: NP_036751.2; *Gallus gallus*: NP_001001298.1; and *Danio rerio*: NP_956845.1. The arrow indicates the location of the changed nucleotide or amino acid.
